# A prospective cohort study on the role of surgical mentorship on medical students’ surgical experience and attitudes towards surgery

**DOI:** 10.1186/s12909-024-06047-0

**Published:** 2024-10-10

**Authors:** Nikki Kerdegari, Edelyne Tandanu, Karen Lee, Rhana Zakri, Prokar Dasgupta, Pankaj Chandak

**Affiliations:** 1 GKT School of Medical Education, King’s College London, London, UK; 2 Guy’s & St Thomas’ NHS Foundation Trust, London, UK; 3 King’s College London, King’s Health Partners, London, UK; 4 Transplant Surgery, London Deanery and Research Fellow, London, UK; 5 School of Immunology and Microbial Sciences, King’s College London, London, UK; 6 Centre for Stem Cells, Regenerative Medicine and Developmental Biology, Institute of Child Health, University College London, London, UK

**Keywords:** Undergraduate, Mentorship, Surgery, Scheme

## Abstract

**Background:**

Exposure to surgery during medical school is limited. We ran a mentorship scheme pairing students at a medical school with local surgeons to improve undergraduate insight. We evaluated the effectiveness of mentorship in increasing surgical exposure and drivers for students seeking surgical mentorship.

**Methods:**

35 surgeons across 7 specialties were recruited and matched with 37 students in years 2–4 for 7 months. Quantitative and qualitative evaluation was done with pre-, mid- and post-scheme questionnaires. Students rated confidence across pursuing a career in surgery, surgical exposure, career insight, understanding the application process, contacts, portfolio development, research knowledge and pursuing extra-curricular activities related to surgery using a 5-point Likert scale. Paired t-tests were performed to measure students’ confidence before and after the scheme.

**Results:**

Of students that completed all 3 questionnaires (*n* = 21), conducting research/audit was most frequently selected as a main goal (81.0%), followed by theatre experience (66.7%) and career progression support (28.6%). The number of students that had assisted in theatre increased by 50.0%. Confidence ratings across all domains increased with 7/8 (87.5%) exhibiting a statistically significant improvement (*p* < 0.05). The greatest improvement was seen in having adequate contacts in surgery from 2.05 to 3.33 (*p* = 0.00). 95.2% would recommend the scheme.

**Conclusions:**

Students gained significantly more surgical experience and were better equipped with the knowledge required to pursue a surgical career. Mentorship schemes are invaluable in supplementing the undergraduate curriculum and empowering students.

**Trial registration:**

Ethical clearance granted by King’s College London Research Ethics Committee, Reference Number MRSU-22/23-34530. .

**Supplementary Information:**

The online version contains supplementary material available at 10.1186/s12909-024-06047-0.

## Background

Within the structured nature of medical school curricula, the challenge of limited surgical experiences among medical students has emerged as a growing concern [1]. The scarcity of opportunities to actively engage with surgery within the undergraduate curriculum can leave students with an insufficient understanding of the intricacies and nuances inherent in surgical practice. Moreover, the inadequacy of surgical education and exposure during medical school serves as a potential deterrent for students to pursue a surgical career; it is well-documented how students formulate negative stereotypes of surgery early into their medical education, particularly on domains surrounding work-life balance, inclusivity and culture within the field [2–7].

Conversely, having early encounters with surgery can serve as a powerful determinant in medical students’ preferences for their future specialty [1, 2, 8–11]. The presence of a strong and positive surgical role model has been documented to dispel preconceived notions held by students, thus supporting them in making well-informed choices when pursuing surgical training [2, 3]. The value of partnering with a surgical mentor is even more evident now, given the rising competitiveness of attaining a surgical training number. The competition ratio for core surgical training has increased from 2.93:1 in 2019 to 4.17:1 in 2023 [12]. The growing competition means aspiring surgeons must strategically tailor their portfolios at an early stage to be shortlisted for interview and subsequently appointed. Mentors play a pivotal role in facilitating this process by offering personalised advice on navigating surgical training and applications, as well as providing valuable research opportunities and theatre experience [13, 14].

Recognising the invaluable role that mentorship can play in guiding and inspiring aspiring surgeons, the King’s College London Surgical Society has taken a proactive step by introducing a surgical mentorship scheme. By pairing students with surgeons from affiliated teaching hospitals, the scheme aims to bridge the gap of limited surgical experience within the medical curriculum by providing students with comprehensive exposure to the field. This encompasses not only direct surgical theatre experience but also a broader perspective on preparing for a surgical career. This includes understanding subspecialty options, managing work-life balance, and addressing essential components of the surgical portfolio. Having recognised the critical position that research output plays in surgical progression [15–17], the scheme has the potential to act as a springboard from which students can gain early involvement with such projects. First piloted in 2018, this scheme is currently in its sixth consecutive year of running. The structure and contents of the programme have continually evolved in response to student feedback to exist in its current form. The 2022/23 mentorship scheme cycle became the basis of this prospective cohort study, which is designed to evaluate the effectiveness of such a mentorship initiative in augmenting surgical exposure for medical students.

The aim of this prospective cohort study is to assess whether surgical mentorship schemes impact medical students’ surgical experience and attitudes towards surgery.

The aims of this study are to evaluate the effectiveness of an undergraduate mentorship scheme in promoting positive attitudes and confidence towards pursuing a surgical career.

## Methods

### Mentor recruitment

The recruitment of mentors to the scheme was managed by the Mentorship Lead and two Mentorship Officers (mentorship team) of the Surgical Society Committee. Surgical trainees and consultants at affiliated teaching hospitals signed up to be mentors via a Google Form. The form included sections for mentors to specify what types of clinical and research opportunities they would be able to offer and how many mentees they would be able to take on. This was advertised via email, posters in non-clinical areas and social media advertisements. During the sign-up process, the purpose of the scheme and role of mentors were clearly outlined. Mentor involvement was incentivised with certificate provision.

### Mentee recruitment

The mentorship scheme was advertised to students via social media and the Society’s monthly newsletter. Medical students in years 2–4, including intercalating medical students, were invited to apply for a place on the scheme via a written application form. Applicants were required to state their aims and goals for the scheme and explain why they would make a good mentee. Applicants also ranked the different hospital sites and surgical specialties and indicated whether they would prefer to be paired with a mentor at their chosen hospital site, a mentor of their chosen surgical specialty or if they had no preference. Due to the oversubscribed nature of the scheme, students applied competitively. All applications were anonymised and scored for quality by two members of the committee. Students were paired to mentors in order of highest scoring application and in line with hospital site/specialty preferencing. Mentors were then emailed the contact details of their mentee and vice versa.

### Induction event

The mentorship team delivered an in-person induction event for mentees. The induction included a presentation from the mentorship team with advice for students on how to maximise the opportunities available to them on the scheme and how to maximise research output during their time with their mentor. Mentees were also provided with a digital handbook outlining the scheme, theatre etiquette, a step-by-step guide on how to perform surgical scrubbing, how to use the e-logbook to record cases and how to get involved in surgical research as a medical student. This event was also an opportunity for mentees to network with each other and meet fellow students who shared an interest in surgery.

### Evaluation

Mentees were emailed pre-, mid- and post-scheme questionnaires (Supplement 1) to allow the mentorship team to monitor mentee aims and subsequent progress during the scheme and explore student perspectives on the qualities of a good mentor. The questionnaire design was informed by feedback received from mentees since the inception of the mentorship scheme in 2018 with the aim to eliminate areas of ambiguity for respondents.

Each questionnaire asked students to rate confidence across eight domains split into three categories – attitudes towards a surgical career, surgical exposure and research experience. The attitudes towards a surgical career category consisted of the domains: pursuing a career in surgery, career insight, understanding the application process, contacts and portfolio development. The surgical exposure category consisted of the single domain of confidence in having adequate exposure to surgery so far in medical school. The research experience domain encompassed two domains of confidence in understanding of audits and pursuing extra-curricular activities and research projects related to surgery.

Students rated confidence across pursuing a career in surgery, surgical exposure, career insight, understanding the application process, contacts, portfolio development, research knowledge and pursuing extra-curricular activities related to surgery using a 5-point Likert scale. Average confidence ratings from the responses are presented as means (± standard deviation), and paired t-tests were performed to measure students’ confidence before and after the scheme, where *p* < 0.05 was considered to be statistically significant. Thematic analysis of free-text responses was also performed.

## Results

### Demographics

A total of 62 students applied to the scheme. 35 surgeons were recruited and matched with 37 students in years 2–4 for 7 months from November 2022 to May 2023. Surgeon mentors came from 7 surgical specialties: trauma and orthopaedics, general surgery, urology, cardiothoracics, hepatobiliary, renal and transplant and ENT. Of the 37 medical students, 21 (56.8%) completed the pre-, mid- and post-scheme questionnaires. Only mentees that completed all three questionnaires were included in the analysis. Of the students who completed all 3 questionnaires, 12 (57.1%) were in year 2, 7 (33.3%) were in year 3 and 2 (9.5%) were intercalating (Table 1).

**Table 1:** Distribution of mentees by year group

Year Group	Number of mentees from the whole mentee cohort (%)	Number of mentees from the cohort of mentees completing all questionnaires (%)
2	18 (48.6)	12 (57.1)
3	10 (27.0)	7 (33.3)
Intercalating	5 (13.5)	2 (9.5)
4	4 (10.8)	0 (0.0)

### Student motivations for surgical mentorship

Thematic analysis revealed that conducting research/audit was most frequently identified as a main goal (81.0%), followed by theatre experience (66.7%) and career progression support (28.6%). 19/21 (90.5%) mentees reported that the mentorship scheme was structured and organised in a way that was helpful to them and the mean Likert rating of how effective the mentorship scheme was in helping them meet the goals they set out to achieve was 3.55 ± 0.86.

At the mid-point of the scheme, commonest themes of work mentees had done towards their goals included research planning (23.8%), conducting research (19.0%) and assisting in theatre (14.3%); and observing in theatre (28.6%), research planning (23.8%) and career advice (19.0%) by the end of the scheme. The most common themes mentees reported for how their mentor could better assist them in achieving their aims and objectives were increased theatre time (28.6%), better communication (23.8%) and research opportunities (23.8%) at the midpoint of the scheme. By the end of the scheme, research opportunities (28.6%), increased frequency of meetings (14.3%) and more opportunities to assist (14.3%) were most commonly reported.

### Effectiveness of the induction events

Prior to the induction event, mean understanding of the mentorship scheme was 2.41 ± 0.80 which increased to 4.32 ± 0.57 after (*p = 0.000*). The most commonly reported takeaways from the induction event were research opportunities for medical students, maximising output from the mentorship scheme and portfolio building, reported by 11 (52.4%), 5 (23.8%) and 5 (23.8%) respectively.

### Frequency of meetings

**Fig. 1:**
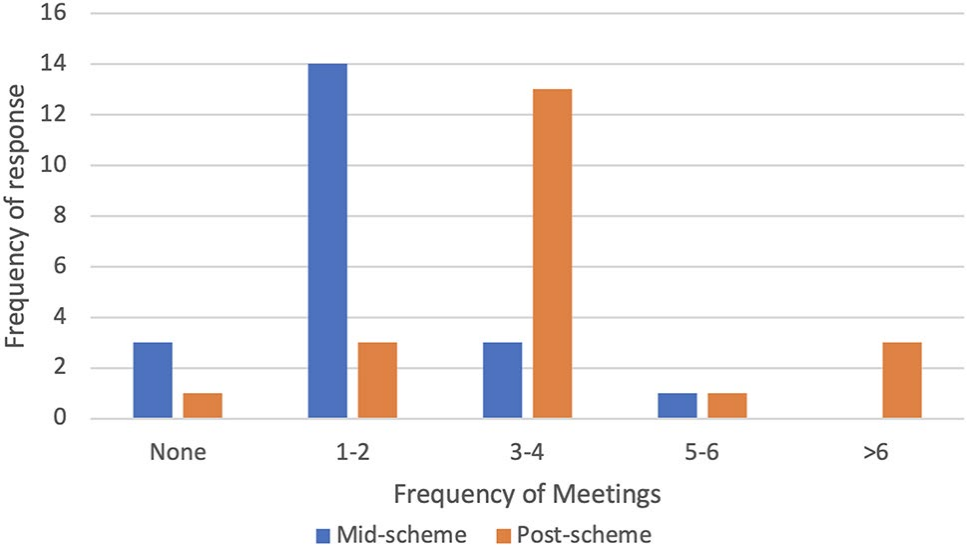
Frequency of meetings with mentor over the course of the scheme

Figure 1 shows the frequency of meetings with mentors over the course of the scheme. In the mid-scheme questionnaire, one student reported difficulties in contacting their mentor, one reported no response from their mentor and the other reported no meetings due to recent reassignment to a new mentor. In the post-scheme questionnaire, one student reported no meetings due to no response from their mentor. Five students reported issues with contacting their mentor or the scheme in general in the mid- and post-scheme questionnaires with all reporting issues relating to communication difficulties.

### Mentee confidence

Mean pre-, mid-, and post-scheme confidence ratings across all domains are shown in Table 2. Mentee confidence was higher at the end of the scheme across every domain assessed with 7/8 (87.5%) of these being statistically significant increases when comparing pre-scheme and post-scheme mean confidence ratings. The largest increase in confidence was seen in mentee confidence in having adequate contacts in their surgical specialty of interest with an increase of 1.28 from 2.05 ± 1.36 to 3.33 ± 1.24 (*p = 0.000*). Mean confidence ratings increased throughout the scheme for every assessed domain apart from confidence in pursuing a surgical specialty after foundation training and pursuing extra-curricular activities and research projects related to surgery which decreased by 0.09 and 0.15 respectively from pre-scheme to mid-scheme. Changes in confidence ratings from pre-scheme to the midpoint of the scheme across all domains were not statistically significant (Supplement 2).

**Table 2:** Mean mentee confidence ratings from pre-, mid- and post-scheme questionnaires

Domain	Mean pre-scheme confidence rating (SD)	Mean mid-scheme confidence rating (SD)	Mean post-scheme confidence rating (SD)	*p* value for paired t-test of pre- and post-scheme confidence
**Attitudes towards a surgical career**				
Pursuing a surgical speciality after foundation training	3.33 (0.86)	3.24 (1.04)	3.95 (0.74)	*p* = 0.006
Understanding of the pros and cons of a career in surgery	3.29 (0.85)	3.57 (1.12)	3.86 (0.73)	*p* = 0.015
Understanding of the application process for surgical training	2.33 (0.73)	2.57 (0.87)	3.52 (1.12)	*p* = 0.000
Having adequate contacts in the surgical specialty they are interested in	2.05 (1.36)	2.38 (0.86)	3.33 (1.24)	*p* = 0.000
Understanding the steps they need to take to improve their surgical portfolio	2.90 (1.09)	3.00 (0.95)	3.76 (1.14)	*p* = 0.001
**Surgical exposure**				
Having adequate exposure to surgery so far in medical school	2.29 (1.00)	2.67 (1.15)	3.14 (1.01)	*p* = 0.002
**Research experience**				
Understanding what an audit cycle involves and how an audit is carried out in hospitals	2.48 (1.36)	2.71 (1.06)	3.05 (1.32)	*p* = 0.104
Pursuing extra-curricular activities and research projects related to surgery	3.29 (1.15)	3.14 (1.01)	3.76 (0.89)	*p* = 0.038

### Attitudes towards a surgical career

Mentee confidence significantly increased across all domains in the attitudes towards a surgical career category. The largest increase in confidence within this domain and across all 8 domains was seen in mentee confidence in having adequate contacts in their surgical specialty of interest with an increase of 1.28 from 2.05 ± 1.36 to 3.33 ± 1.24 (*p = 0.000*).

### Surgical exposure

**Fig. 2:**
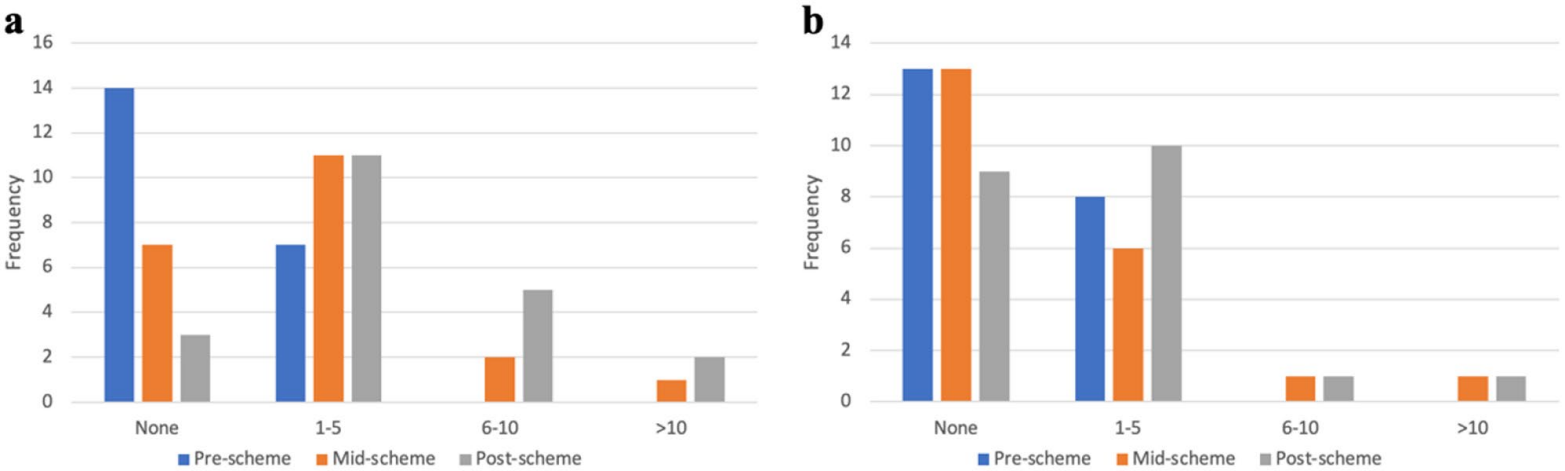
Frequency of cases **a** observed, **b** assisted in

Mentee confidence in having adequate exposure to surgery during medical school displayed a statistically significant increase from 2.29 ± 1.00 to 3.14 ± 1.01 (*p = 0.002).* Furthermore, the number of students that had assisted in theatre increased by 50% from 8/21 (38.1%) before the scheme. Before starting the scheme, 13 (61.8%) had never assisted and 8 (38.1%) had assisted 1–5 times. By the end of the scheme, 9 (42.9%) had not assisted, 10 (47.6%) had assisted 1–5 times, 1 (4.8%) had assisted 6–10 times and 1 (4.8%) had assisted > 10 times. Figure 2 shows the volume of cases students observed and assisted in during the scheme.

### Research experience

**Fig. 3:**
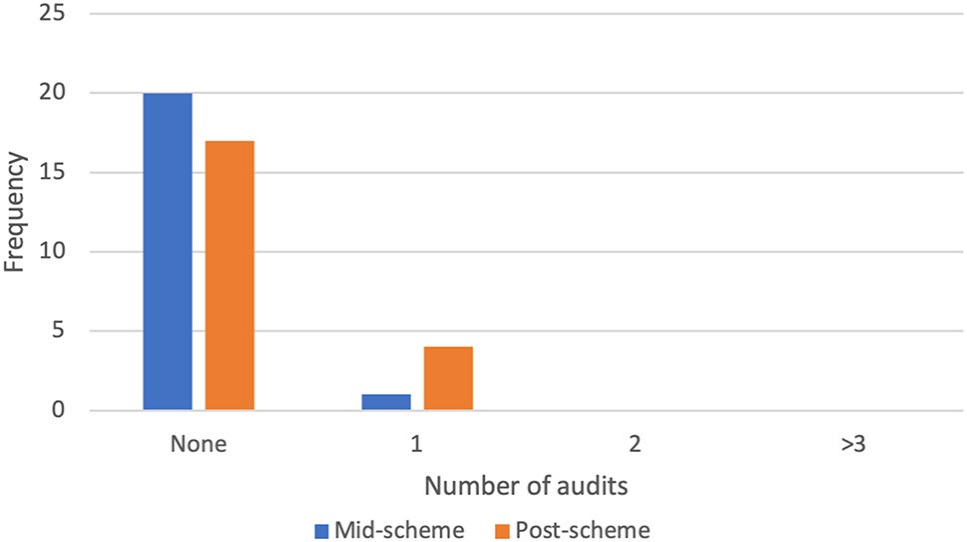
The number of audits that students carried out during the scheme

The only domain to not exhibit a statistically significant increase from pre-scheme to post-scheme analysis was confidence in understanding of audits which increased from 2.48 ± 1.36 to 3.05 ± 1.32 (*p = 0.104)*. Before starting the scheme, 19 (90.5%) students had not carried out an audit and 2 (9.5%) had previously carried out one audit. Figure 3 shows the number of audits that students carried out during the scheme.

Confidence in pursuing extra-curricular activities and research projects related to surgery significantly increased from 3.29 ± 1.15 to 3.76 ± 0.89 (*p = 0.038*). Prior to the scheme, 2 (9.5%) students had previously delivered oral presentations, one presented once and the other had presented twice. During the course of the scheme, no students presented any research by the mid-point of the scheme and by the end of the scheme, one student had presented on a local departmental level. Additionally, before starting the scheme, 2 (9.5%) students had previously published, one had one publication and the other had two. Over the scheme, no student had published. 

### Attributes of a good undergraduate surgical mentor

**Fig. 4:**
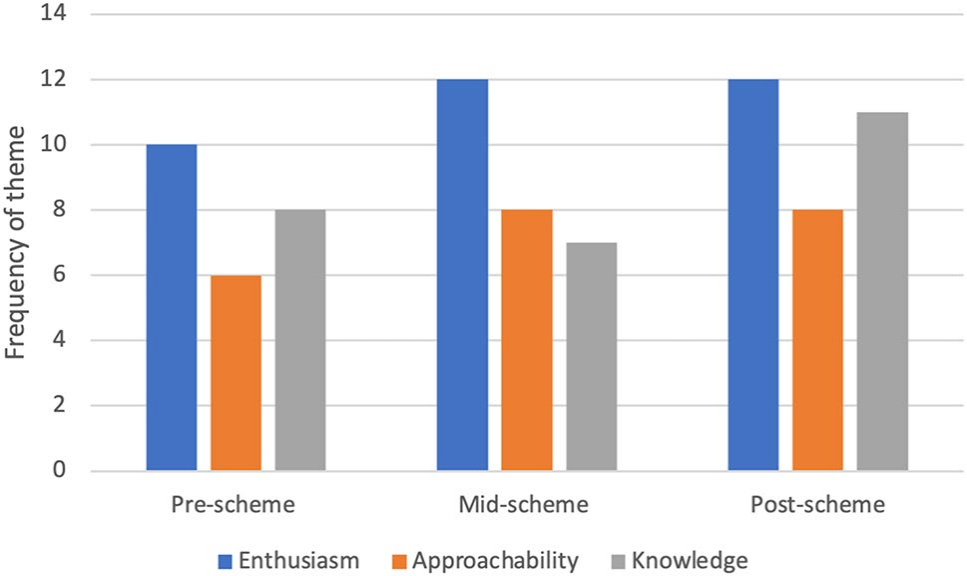
Frequency of commonest themes of top qualities of a mentor over time

Thematic analysis of free-text mentee responses revealed students perceived enthusiasm, approachability and knowledge to be the top qualities of a mentor (Fig. 4).

Gaining a realistic insight into what a career in surgery entails (38.1%), learning surgical skills (14.3%) and theatre experience (14.3%) were identified as the most valuable insights/ lessons students gained from their mentors. Aspects of the scheme that students found most helpful included surgical exposure (38.1%), conducting research with their mentor (19.0%), having a named surgical contact to be able to ask questions and build a professional working relationship with (14.3%) and shadowing (14.3%).

19 (90.5%) agreed that the mentorship programme was structured and organised in a way that was helpful for them. 20/21 (95.2%) of students said they would recommend the mentorship scheme to others. The one student who did not recommend the mentorship scheme to others was not able to meet their mentor at all during the scheme. This lack of interaction significantly impacted their experience and resulted in them not recommending the scheme. The student was then re-enrolled in the 2023/24 cycle, and the following positive experience with a new mentor led to them recommending the scheme in the subsequent end-of-scheme questionnaire.

**Fig. 5:**
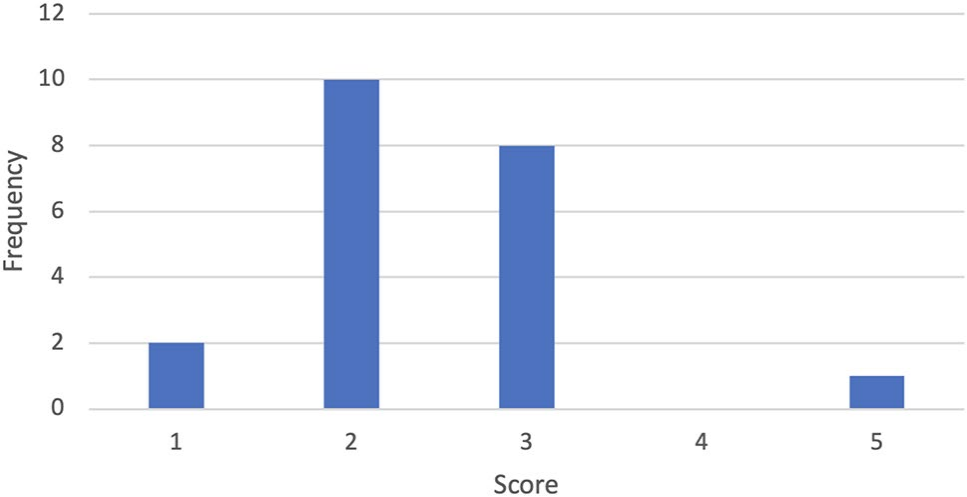
Frequencies of Likert ratings given to question “How effective was the mentorship scheme in helping you to meet the goals you set out to achieve?”

Mean Likert rating of how effective the mentorship scheme was in helping mentees meet the goals they set out to achieve was 3.57 ± 0.87. Frequencies of each rating are shown in Fig. 5. A variety of unique individualised answers were given to the question ‘What would you have done differently if given the chance to participate in the scheme again?’ however some recurring themes included undertaking research (19.0%), meeting their mentor more regularly (9.5%), being more proactive (9.5%) and seeking out exposure to more surgical specialties (9.5%).

## Discussion

This study investigated the effectiveness of a 7-month long mentorship scheme in promoting attitudes and confidence towards pursuing a surgical career. Analysis of mentee responses revealed statistically significant increases across all but one domain, most notably, having adequate contacts in their specialty of interest. There were no notable changes in confidence from pre-scheme to the midpoint of the scheme (Supplement 2). However, significant developments were seen by the scheme’s conclusion, therefore evidencing that our scheme was of a suitable duration. From our results, we can conclude that students gained significantly more surgical experience and were better equipped with the knowledge required to pursue a surgical career, as compared to when they first enrolled in the scheme.

We opted to use our own non-validated questionnaire to evaluate our scheme. We acknowledge that the use of non-validated questionnaires does limit the generalisability of our findings. Whilst they have not been formally evaluated, the series of questionnaires used in this study have been developed following the running of this mentorship scheme five years prior to the 2022/23 cycle. It has been adapted following pilot testing and feedback in past iterations of our scheme over the preceding five years aiming to eliminate areas of ambiguity for respondents. A number of validated tools for evaluating mentorship exist. However, common issues we encountered with such tools included tools intended for non-clinician educator mentors [18] and tools designed for postgraduate academic mentorship [19]. The medical student mentoring evaluation tool, the Munich-Evaluation-of-Mentoring-Questionnaire (MEMeQ) is a validated questionnaire for evaluating satisfaction with mentoring relationships in medical education [20]. This tool requires mentees to define individual areas of interest in their mentoring relationship, then assign relative levels of personal importance to them and finally rate their individual level of satisfaction with their mentors’ support in each area of interest. This tool was not suitable for our study as our scheme had pre-determined areas for mentor-mentee pairs to focus on for the duration of the scheme.

The Medical Student Scholar-Ideal Mentor Scale (MSS-IMS) is a validated tool for assessing mentors for medical student research projects [21]. We did not opt to use this tool given its specific area of focus to only one of the activities of our mentorship scheme. However, as undertaking research/audit was most commonly selected as the main goal for our scheme and the only domain to not exhibit a statistically significant increase from pre-scheme to post-scheme analysis was confidence in understanding of audits, this tool could be implemented as an additional component of our scheme analysis. This will provide a validated method to identify current limitations of this particular goal and learning points to support mentors in mentoring their students with regards to undertaking research specifically.

Appreciating the pivotal role of research in surgical training, our scheme aims to introduce students to research involvement. Despite conducting research/audit being indicated as the main goal, it was the only domain in which a statistically significant improvement was not achieved. This was substantiated by feedback collected where mentees highlighted the lack of research/audit experience as an unmet goal. Mid- and post-scheme questionnaires included questions on whether students had delivered poster/oral presentations or published research. However, all mentees answered no to these questions apart from one departmental level presentation. This may be explained by the 7-month duration of the scheme, a timeframe that may be too short for completion of a research project, particularly by a student without prior experience. Analysis of research knowledge and output from the scheme could be better assessed by longer term follow up with additional questionnaires at one year and two years post-scheme. Future pre-scheme questionnaires used for this mentorship scheme could include free-text questions to improve data gathered on students’ motivations for applying and allow identification of their challenges to date to identify themes and subsequent changes to the scheme to improve mentee accessibility to surgery. The current questionnaires could also be improved with the addition of free-text questions for students to report the types of research they have been undertaking with their mentor and highlight any limitations. This would be the first step in identifying what changes are needed to the scheme to better support mentees’ research aspirations. Mentors proposing specific research projects during the mentor sign up process, for example as part of their own existing active research initiatives, could help support students wishing to pursue research by avoiding delays in starting projects, thereby ensuring this goal is met. Other challenges that our mentees identified were a lack of engagement from mentors and clashing schedules, which inevitably led to lesser learning opportunities. Each mentor-mentee pair were instructed to meet on at least 3 occasions with both parties informed of this arrangement before applying to the scheme and told to negotiate suitable timings to achieve this. Other schemes have utilised online booking systems and surgeon administrative assistants [3, 22]. In addition to providing accountability, such systems simultaneously act as a method for the mentorship team to monitor engagement in real time and allow for earlier troubleshooting of any potential problems.

In our scheme, mentor-mentee matching was solely based on the mentee’s preference of specialty or hospital site. Studies looking at gender-concordant mentorship, particularly between women, have shown huge success and influence on their interest in pursuing a surgical career, realising their professional goals, and increasing exposure to positive mentors and role models [1, 4, 23]. Other studies have shown gender disparities within surgery, with female students/trainees reporting less opportunities for learning and career progression compared to their male counterparts [24, 25]. This highlights the importance of addressing this issue by introducing gender-based pairing in future schemes.

With a total of 61 applications and only 37 available positions, the selection process was highly competitive. This presents a barrier for students seeking such mentorship opportunities. It is therefore imperative that future schemes focus on expanding mentor recruitment as a priority to meet student demand. A novel approach to tackling oversubscription of such mentorship schemes is using online platforms, such as Proximie, allowing mentees to virtually observe surgeries in real time [26]. Live-streaming surgery has been found to successfully increase student knowledge and a well-accepted mode of undergraduate surgical education [27].

Another avenue in which we could enhance the effectiveness of our scheme would be the introduction of formal training for mentors, such as the Health Education England online training programme [28]. This ensures uniformity in the quality and delivery of mentorship. A foreseeable challenge in implementing this intervention is potential resistance in its uptake from mentors, who may view it as an additional demand. Further research on the efficacy of such interventions within mentorship is needed to assess the true value of mentor training.

Taking on the voluntary role of a mentor, on top of an already busy rota, is incredibly demanding. Our feedback indicates that mentee experiences and learning are negatively impacted by mentors’ limited engagement, often due to their heavy workloads. This suggests that mentors are less valuable to their mentees when overwhelmed. This could be overcome by hospital trusts or medical schools allocating dedicated time for surgeons to act as mentors, in a similar way to being an educational supervisor. Institutional support of mentoring efforts may play a role in incentivising more surgeons to participate as mentors. Additionally, the concept of co-regulation for successful mentorship relationships, whereby mutual investment contributes to mutual learning and growth, should be emphasised to mentors [29] to educate them on the benefits they themselves can gain whilst mentoring students. Alternatively, speed mentoring has proven successful for surgical trainees and has potential for undergraduate mentorship, being less time-intensive than traditional mentorship programmes [30]. Whilst we acknowledge that undergraduate mentorship is a significant and enduring commitment, on both the organising team and mentors’ part, we firmly advocate for its importance in shaping tomorrow’s surgical workforce. This is further supported by research demonstrating that students who had undergone mentorship have statistically significant advancements in their career progression over peers who lack such support [31].

Our study faced several limitations, most notably the small sample size. Additionally, most respondents were Year 2 students, in their first year of clinical placements. This provides a limited perspective and may not be representative of the broader student population. The fact that our participants are a self-selected group also introduces inherent bias in our results, as they are more likely to be more motivated and interested in surgery than the average student. This scheme is run as a supplementary opportunity to the requirements of the undergraduate curriculum, reflective of the fact that this initiative is driven by the medical school’s student-led surgical society. We advertised mentee participation across multiple platforms (email, posters and social media), aiming to reach to all medical students and not just those with an existing interest in surgery. Mentorship schemes have been integrated into existing undergraduate clinical placements [32, 33] and we could look to integrate our mentorship scheme into existing surgical placements to allow all students to have a mentor. Unfortunately, only 21 students completed all 3 questionnaires. Questionnaire response rate may be improved by implementing mandatory in-person sessions at the midpoint and end of the scheme. Such sessions would provide touchpoints for questionnaire completion and provide an additional medium for mentees to raise concerns or troubleshoot in addition to the existing email inbox run by the mentorship team. Moreover, data was collected immediately after the conclusion of the scheme, limiting our ability to evaluate the long-term impact of our scheme on mentees. Finally, we did not conduct a formal evaluation of mentors’ experience of the scheme. Future cohorts will have the addition of a post-scheme mentor questionnaire with a combination of free-text and nominal questions to assess mentee performance from the mentor perspective and identify mentors’ perceived benefits and limitations of participation in the scheme. Furthermore, the current approach could be expanded by offering additional incentives and implementing measures such as more frequent reminders as deadlines for questionnaire submission approach.

The recent introduction of the Medical Licensing Assessment with its surgical syllabus [34] and the Royal College of Surgeons of England National Undergraduate Curriculum for Surgery [35] are two tools that could be integrated into mentorship schemes to provide further standardisation for mentees. These surgical curricula aids can serve study resources to help equip mentees with the appropriate knowledge when preparing for theatre lists to maximise learning during surgical experiences with mentors. Future mentorship schemes should integrate these curricula into their design to enhance the quality of education and preparation mentees receive. A set of basic surgical science questions could be added to objectively assess mentee’s understanding of core surgical content before, during and after the scheme. Mentors could also provide feedback at post-scheme evaluation on their mentee’s knowledge and skill development. Mentors should also be signposted to the AMEE guidance on mentoring [36] at the start of such schemes to support them in their role .

## Conclusion

Following completion of this 7-month mentorship scheme, students gained significantly more surgical experience and were better equipped with the knowledge required to pursue a surgical career with an improvement in their confidence. The scheme offered networking opportunities, theatre experience and clinical insight; ultimately acting as a springboard from which students can seek out further opportunities within surgery. Consequently, mentorship schemes are invaluable in supplementing the undergraduate curriculum and empowering students. Future schemes should prioritise securing research project proposals from the early stages of the scheme in order to better support mentees in building their research skillset through participation in projects. Further research is required to provide long-term follow up on individuals undertaking such mentorship schemes as students and the subsequent choice of specialty they pursue.

## Electronic supplementary material

Below is the link to the electronic supplementary material.

Supplementary Material 1

Supplementary Material 2

## Data Availability

The anonymised datasets used during the current study are available from the corresponding author on reasonable request.
