# Supplementary material for: A prospective cohort study on the role of surgical mentorship on medical students’ surgical experience and attitudes towards surgery
Source: BMC Med Educ. 2024 Oct 10;24:1116. doi: 10.1186/s12909-024-06047-0 (PMC11468475; doi:10.1186/s12909-024-06047-0)
Supplement: Supplementary file 1 — Supplementary Material 1 [file 12909_2024_6047_MOESM1_ESM.pdf]

Mentee Pre-Mentorship Questionnaire 2022-23

Dear mentees,

Thank you for signing up to the Mentorship Scheme and for completing this questionnaire! Any information we can collect throughout the scheme is extremely useful to monitor both how you are progressing and how we can improve the scheme in the future. We wish you all the best for the scheme ahead and hope you have a positive experience with your mentor!

If you have any questions, please do not hesitate to contact us at kclmentorship@gmail.com.

This year KCL Surgical Society is conducting research on the mentorship scheme which we would like to invite you to participate in. Please read the following information sheet about the research project.

INFORMATION SHEET FOR PARTICIPANTS ***Version 01, 09/11/22***
Ethical Clearance Reference Number MRSU-22/23-34530

You can access a PDF copy of this information sheet for your own record-keeping
via https://drive.google.com/file/d/1kP6SOrT0thfazTgYknRRYDzi-I-1GhgS/view?usp=sharing

The role of surgical mentorship on medical students' surgical experience and attitudes towards surgery

Invitation Paragraph

I would like to invite you to participate in this research project which forms part of KCL Surgical Society's research into its academic initiatives. Before you decide whether you want to take part, it is important for you to understand why the research is being done and what your participation will involve. Please take time to read the following information carefully and discuss it with others if you wish. Ask me if there is anything that is not clear or if you would like more information.

What is the purpose of the project?

The purpose of the project is to assess whether surgical mentorship schemes impact medical students' surgical experience and attitudes towards surgery. The project aims to explore the reasons why medical students choose to participate in surgical mentorship schemes; to assess how medical students' surgical exposure, research experience and attitudes towards a surgical career are affected by mentorship schemes; and to identify the attributes that make a good undergraduate surgical mentor and formulate recommendations on how other centres can implement similar schemes.

Why have I been invited to take part?

You are being invited to participate in this project because you are a medical student at King's College London that has successfully gained a place as a mentee on the KCL Surgical Society Mentorship Scheme.

What will happen if I take part?

If you choose to take part in the project your questionnaires that you complete as part of your time on the mentorship scheme (the pre-mentorship questionnaire, mid-mentorship questionnaire and post-mentorship questionnaire) will be analysed. Participation will take place via Google Forms sent out in November, February and May. As part of participation, you will be asked to answer a set of questions about your experience thus far on the scheme to allow conclusions to be drawn about the value of undergraduate surgical mentorship schemes. The questionnaires will contain the following personal information: your full name, K number and email address.

Do I have to take part?

Participation is completely voluntary. You should only take part if you want to and choosing not to take part will not disadvantage you in any way. Once you have read the information sheet, please contact us if you have any questions that will help you make a decision about taking part The pre-mentorship questionnaire will ask if you consent to taking part and subsequent questionnaires will remind you of the study and that you can withdraw if you wish.

What are the possible risks of taking part?

There are no risks associated with taking part.

What are the possible benefits of taking part?

There are no intended benefits for individual participants.

Data handling and confidentiality

Your data will be processed under the terms of UK data protection law (including the UK General Data Protection Regulation (UK GDPR) and the Data Protection Act 2018).

- The personal data collected (name, K number and email address) will be stored in an encrypted and password protected Excel spreadsheet saved to KCL OneDrive while data collection and analysis is ongoing.

- Once all 3 survey responses have been collected and analysed, your data will be fully anonymised.

- In line with the College's Records and Data Retention Schedule, after data has been collected it will be retained for 5 years following publication.

- Data will only be shared within the research team.

King's College London has a responsibility to keep information collected about you safe and secure, and to ensure the integrity of research data. Specialist teams within King's College London continually assess and ensure that data is held in the most appropriate and secure way.

Data Protection Statement

If you would like more information about how your data will be processed under the terms of UK data protection laws please visit the link below:

https://www.kcl.ac.uk/research/support/research-ethics/kings-college-london-statement-on-use-of-personal-data-in-research

What if I change my mind about taking part?

You are free withdraw at any point of the project, without having to give a reason. Withdrawing from the project will not affect you in any way. You are able to withdraw your data from the project up until 31/05/22, after which withdrawal of your data will no longer be possible as by this point the data will have been anonymised and committed to the final report. If you choose to withdraw from the project, we will not retain the information you have given thus far.

What will happen to the results of the project?

The results of the project will be summarised in an article and will be published in a medical journal. The published article will be circulated with participants should they wish to read it.

Who should I contact for further information?

If you have any questions or require more information about this project, please contact me using the following contact details: nikki.kerdegari@kcl.ac.uk

What if I have further questions, or if something goes wrong?

If this project has harmed you in any way or if you wish to make a complaint about the conduct of the project you can contact King's College London using the details below for further advice and information:

Project supervisor: Professor Nicki Cohen, nicki.cohen@kcl.ac.uk

Thank you for reading this information sheet and for considering taking part in this research.

kclmentorship@gmail.com Switch account

Not shared

* Indicates required question

Do you consent to anonymous data from your Mentorship Scheme questionnaires to be used for research? By ticking yes, you consent to the following: *

1. I confirm that I have read and understood the information sheet dated 09/11/22, Version 01 for the above project. I have had the opportunity to consider the information and asked questions which have been answered to my satisfaction.

2. I consent voluntarily to be a participant in this project and understand that I can refuse to take part and can withdraw from the project at any time, without having to give a reason, up until 31/05/22

3. I consent to the processing of my personal information for the purposes explained to me in the Information Sheet. I understand that such information will be handled under the terms of UK data protection law, including the UK General Data Protection Regulation (UK GDPR) and the Data Protection Act 2018.

4. I understand that my information may be subject to review by responsible individuals from the College for monitoring and audit purposes.

5. I understand that confidentiality and anonymity will be maintained, and it will not be possible to identify me in any research outputs

6. I understand that the information I have submitted will be published as a report

7. I agree to be re-contacted in the future by King's College London researchers regarding this project.

8. I consent to my identifiable data being stored in a password protected Excel spreadsheet saved to KCL One Drive as described in the information sheet.

○ Yes

○ No

Next

Clear form

Feedback on the Induction Event

We hope you found the Induction Event informative and enjoyed the session!

As this is our first time running such an event, it would be really helpful for us to know how beneficial you found it and what improvements we can make to better address your needs!

On a scale of 1 to 5, how would you rate your understanding of the mentorship scheme ***BEFORE*** the induction event? *

(*1* = very poor ; *5* = excellent)

1	2	3	4	5
○	○	○	○	○

On a scale of 1 to 5, how would you rate your understanding of the mentorship scheme ***AFTER*** the induction event? *

(*1* = very poor ; *5* = excellent)

1	2	3	4	5
○	○	○	○	○

How did you find the duration of the session? *

○ Too long

○ Just right

○ Too short

What is your biggest takeaway from the Induction Event? *

Your answer

What do you think could be improved about the session? *

Your answer

Back Next Clear form

Please rate your confidence in the following on a scale of 1-5

1 - Not at all confident
2 - Slightly confident
3 - Moderately confident
4 - Quite confident
5 - Extremely confident

1. Pursuing a surgical speciality after foundation year training. *

1	2	3	4	5
○	○	○	○	○

2. Having adequate exposure to surgery so far in medical school *

1	2	3	4	5
○	○	○	○	○

3. Understanding of the pros and cons of a career in surgery *

1	2	3	4	5
○	○	○	○	○

4. Understanding of the application process for surgical training *

1	2	3	4
○	○	○	○

\

5. Having adequate contacts in the surgical speciality that I am interested in *

1	2	3	4	5
○	○	○	○	○

6. Understanding the steps that I need to take to improve my surgical portfolio *

1	2	3	4	5
○	○	○	○	○

7. Understanding what an audit cycle involves and how an audit is carried out in hospitals *

1	2	3	4	5
○	○	○	○	○

8. Pursuing extra-curricular activities and research projects related to surgery *

1	2	3	4	5
○	○	○	○	○

Back   Next   Clear form

Closed questions

A disclaimer from the Mentorship Scheme committee: the questions below are for us to get an overall idea of the cohort, so don't worry if you haven't done any of the below!

1. Have you previously observed any surgeries? If yes, how many times? *

- ◯ None
- ◯ 1-5
- ◯ 6-10
- ◯ >10

2. Have you previously assisted in surgery? If yes, how many times? *

- ◯ None
- ◯ 1-5
- ◯ 6-10
- ◯ >10

3. Have you previously carried out an audit? If yes, how many? *

- ◯ None
- ◯ 1
- ◯ 2
- ◯ >3

4. Have you presented an oral presentation or a poster? If yes, how many and which type? *

Your answer

5. Have you published any publications? If yes, how many? (Please list if you can) *

Your answer

Back   Next   Clear form

Open Questions

These answers will be sent to your mentor, please try to answer these in as much detail as you can. This will help us give some guidance to your mentors on what areas you would like to focus on. Thank you!

1. In your opinion, what are the top 3 qualities of a successful mentor? *

Your answer

2. What do you hope to achieve at the end of this scheme? Please list up to 3 aims/objectives. *

Your answer

3. What specific questions would you like answered by your mentor? Please list up to 3. *

Your answer

Back Next Clear form

Thank you!

Should you have any questions/concerns throughout the scheme, please do not hesitate to contact us at kclmentorship@gmail.com. We hope you enjoy and make the most out of your time on this scheme, happy learning!

Best wishes,
KCL Mentorship Team :)

Mentee Mid-Mentorship Questionnaire 2022-23

Dear mentees,

We hope you've been enjoying your time and full use of the Mentorship Scheme thus far! Any information we can collect throughout the scheme is extremely useful to monitor both how you are progressing and how we can improve the scheme in the future. We wish you all the best for the scheme ahead and hope you have a positive experience with your mentor!

If you have any questions, please do not hesitate to contact us at kclmentorship@gmail.com.

This year KCL Surgical Society is conducting research on the mentorship scheme. Just a reminder that if you consented to the study on the Pre-Mentorship questionnaire form, data from this form will also be collected for research purposes. Please read the following information sheet, which contains the full study information about our research project, should you wish to find out more information.

INFORMATION SHEET FOR PARTICIPANTS *Version 01, 09/11/22*
Ethical Clearance Reference Number MRSU-22/23-34530

You can access a PDF copy of this information sheet for your own record-keeping via https://drive.google.com/file/d/1kP6SOrT0thfazTgYknRRYDzi-I-1GhgS/view?usp=sharing
The role of surgical mentorship on medical students' surgical experience and attitudes towards surgery

Who should I contact for further information?

If you have any questions or require more information about this project, please contact me using the following contact details: nikki.kerdegari@kcl.ac.uk

kclmentorship@gmail.com Switch account

Not shared

* Indicates required question

Full name *

Your answer

K number *

Your answer

KCL email address *

Your answer

Next

Clear form

Please rate your confidence in the following on a scale of 1-5

1 - Not at all confident
2 - Slightly confident
3 - Moderately confident
4 - Quite confident
5 - Extremely confident

1. Pursuing a surgical speciality after foundation year training. *

1	2	3	4	5
○	○	○	○	○

2. Having adequate exposure to surgery so far in medical school *

1	2	3	4	5
○	○	○	○	○

3. Understanding of the pros and cons of a career in surgery *

1	2	3	4	5
○	○	○	○	○

4. Understanding of the application process for surgical training *

1	2	3	4	5
○	○	○	○	○

5. Having adequate contacts in the surgical speciality that I am interested in *

1	2	3	4	5
○	○	○	○	○

6. Understanding the steps that I need to take to improve my surgical portfolio *

1	2	3	4	5
○	○	○	○	○

7. Understanding what an audit cycle involves and how an audit is carried out in hospitals *

1	2	3	4	5
○	○	○	○	○

8. Pursuing extra-curricular activities and research projects related to surgery *

1	2	3	4	5
○	○	○	○	○

Back Next Clear form

Closed questions

A disclaimer from the Mentorship Scheme committee: the questions below are for us to get an overall idea of the cohort, so don't worry if you haven't done any of the below!

1. Since joining the Mentorship Scheme, how many surgeries have you observed? *

- ○ None
- ○ 1-5
- ○ 6-10
- ○ >10

2. Since joining the Mentorship Scheme, how many surgeries have you assisted in? *

- ○ None
- ○ 1-5
- ○ 6-10
- ○ >10

3. Since joining the Mentorship Scheme, have you carried out an audit? If yes, how many? *

- ○ None
- ○ 1
- ○ 2
- ○ >3

4. Since joining the Mentorship Scheme, have you presented an oral presentation or a poster? If yes, how many and which type? *

Your answer

5. Since joining the Mentorship Scheme, have you published any publications? If yes, how many? (Please list if you can) *

Your answer

6. Have you had any issues with contacting your mentor or with the mentorship scheme in general?

- ◯ Yes
- ◯ No

If yes, please share about this in more detail.

Your answer

7. How many meetings have you had with your mentor thus far? *

- ◯ None
- ◯ 1-2
- ◯ 3-4
- ◯ 5-6
- ◯ >6

If your answer to the previous question was none, please indicate the reason for this.

Your answer

Back Next Clear form

Open Questions

These updated answers will be sent to your mentor, please try to answer these in as much detail as you can. This will help us give some guidance to your mentors on what areas you would like to focus on. Thank you!

1. In your opinion, what are the top 3 qualities of your mentor? *

Your answer

2. In the previous form, you listed up to 3 aims/objectives to you were hoping to achieve. What have you and your mentor done to work towards these goals? *

Your answer

3. What specific questions would you like answered by your mentor? Please list up to 3. *

Your answer

4. List 3 ways in which your mentor can better assist you in achieving these aims/objectives? *

Your answer

Back Next Clear form

Mentee End-Mentorship Questionnaire 2022-23

Dear mentees,

We hope you've been enjoying your time and full use of the Mentorship Scheme thus far! As this year's scheme draws to an end, we hope to learn more about your experience on the scheme. Any information we can collect throughout the scheme is extremely useful to monitor both how you are progressing and how we can improve the scheme in the future. We wish you all the best for the scheme ahead and hope you have a positive experience with your mentor!

If you have any questions, please do not hesitate to contact us at kclmentorship@gmail.com.

This year KCL Surgical Society is conducting research on the mentorship scheme. Just a reminder that if you consented to the study on the Pre-Mentorship questionnaire form, data from this form will also be collected for research purposes. Please read the following information sheet, which contains the full study information about our research project, should you wish to find out more information.

INFORMATION SHEET FOR PARTICIPANTS *Version 01, 09/11/22*
Ethical Clearance Reference Number MRSU-22/23-34530

You can access a PDF copy of this information sheet for your own record-keeping via https://drive.google.com/file/d/1kP6SOrT0thfazTgYknRRYDzi-l-1GhgS/view?usp=sharing
The role of surgical mentorship on medical students' surgical experience and attitudes towards surgery

Who should I contact for further information?

If you have any questions or require more information about this project, please contact me using the following contact details: nikki.kerdegari@kcl.ac.uk

kclmentorship@gmail.com Switch account

Not shared

* Indicates required question

Full name *

Your answer

K number *

Your answer

KCL email address *

Your answer

Next

Clear form

Please rate your confidence in the following on a scale of 1-5

1 - Not at all confident
2 - Slightly confident
3 - Moderately confident
4 - Quite confident
5 - Extremely confident

1. Pursuing a surgical speciality after foundation year training. *

1	2	3	4	5
○	○	○	○	○

2. Having adequate exposure to surgery so far in medical school *

1	2	3	4	5
○	○	○	○	○

3. Understanding of the pros and cons of a career in surgery *

1	2	3	4	5
○	○	○	○	○

4. Understanding of the application process for surgical training *

1	2	3	4	5
○	○	○	○	○

5. Having adequate contacts in the surgical speciality that I am interested in *

1	2	3	4	5
○	○	○	○	○

6. Understanding the steps that I need to take to improve my surgical portfolio *

1	2	3	4	5
○	○	○	○	○

7. Understanding what an audit cycle involves and how an audit is carried out in hospitals *

1	2	3	4	5
○	○	○	○	○

8. Pursuing extra-curricular activities and research projects related to surgery *

1	2	3	4	5
○	○	○	○	○

Back Next Clear form

Closed questions

A disclaimer from the Mentorship Scheme committee: the questions below are for us to get an overall idea of the cohort, so don't worry if you haven't done any of the below!

1. Since joining the Mentorship Scheme, how many surgeries have you observed? *

- ◯ None
- ◯ 1-5
- ◯ 6-10
- ◯ >10

2. Since joining the Mentorship Scheme, how many surgeries have you assisted in? *

- ◯ None
- ◯ 1-5
- ◯ 6-10
- ◯ >10

3. Since joining the Mentorship Scheme, have you carried out an audit? If yes, how many? *

- ◯ None
- ◯ 1
- ◯ 2
- ◯ >3

4. Since joining the Mentorship Scheme, have you presented an oral presentation or a poster? If yes, how many and which type? *

Your answer

5. Since joining the Mentorship Scheme, have you published any publications? If yes, how many? (Please list if you can) *

Your answer

6. Have you had any issues with contacting your mentor or with the mentorship scheme in general?

- ◯ Yes
- ◯ No

If yes, please share about this in more detail.

Your answer

7. How many meetings have you had with your mentor thus far? *

- ◯ None
- ◯ 1-2
- ◯ 3-4
- ◯ 5-6
- ◯ >6

If your answer to the previous question was none, please indicate the reason for this.

Your answer

Back Next Clear form

Open Questions

These updated answers will be sent to your mentor, please try to answer these in as much detail as you can. This will help us give some guidance to your mentors on what areas you would like to focus on. Thank you!

1. In your opinion, what are the top 3 qualities of your mentor? *

Your answer

2. In the previous form, you listed up to 3 aims/objectives to you were hoping to achieve. What have you and your mentor done to work towards these goals? *

Your answer

3. What specific questions would you like answered by your mentor? Please list up to 3. *

Your answer

4. List 3 ways in which your mentor can better assist you in achieving these aims/objectives? *

Your answer

Back Next Clear form

Reflections on the Mentorship Scheme

This section focuses on what you have learnt during the scheme and why/how you found it beneficial. Please be as candid as possible, it would greatly help us to troubleshoot and improve the scheme in the future.

Was the mentorship program structured and organized in a way that was helpful for you? *

- ◯ Yes
- ◯ No

If your answer was no, what changes could have been made to benefit you more? *

(If you answered yes, please respond with "NIL".)

Your answer

How effective was the mentorship scheme in helping you to meet the goals you set out to achieve? *

	1	2	3	4	5	
Not helpful at all	◯	◯	◯	◯	◯	Very helpful

What were some of the most valuable insights or lessons that you gained from your mentor? (Please list up to 3, you can respond in a bullet point format) *

Your answer

Which aspects of the scheme did you find the most helpful? (Please list at least 3, you can respond in a bullet point format) *

Your answer

What would you have done differently if given the chance to participate in the scheme again? (Please list up to 3, you can respond in a bullet point format) *

Your answer

Would you recommend this mentorship program to others? If yes, why? If not, why not? *

Your answer

Back   Next   Clear form
