# Supplementary material for: A prospective cohort study on the role of surgical mentorship on medical students’ surgical experience and attitudes towards surgery
Source: BMC Med Educ. 2024 Oct 10;24:1116. doi: 10.1186/s12909-024-06047-0 (PMC11468475; doi:10.1186/s12909-024-06047-0)
Supplement: Supplementary file 2 — Supplementary Material 2 [file 12909_2024_6047_MOESM2_ESM.docx]

*Supplementary table of paired t-tests of pre- and mid-scheme confidence ratings*

| **Domain** | **Mean pre-scheme confidence rating (SD)** | **Mean mid-scheme confidence rating (SD)** | **p value for paired t-test of pre- and mid-scheme confidence** |
| --- | --- | --- | --- |
| Pursuing a surgical speciality after foundation training | 3.33 (0.86) | 3.24 (1.04) | p=0.733 |
| Understanding of the pros and cons of a career in surgery | 3.29 (0.85) | 3.57 (1.12) | p=0.300 |
| Understanding of the application process for surgical training | 2.33 (0.73) | 2.57 (0.87) | p=0.329 |
| Having adequate contacts in the surgical specialty they are interested in | 2.05 (1.36) | 2.38 (0.86) | p=0.232 |
| Understanding the steps they need to take to improve their surgical portfolio | 2.90 (1.09) | 3.00 (0.95) | p=0.724 |
| Having adequate exposure to surgery so far in medical school | 2.29 (1.00) | 2.67 (1.15) | p=0.278 |
| Understanding what an audit cycle involves and how an audit is carried out in hospitals | 2.48 (1.36) | 2.71 (1.06) | p=0.479 |
| Pursuing extra-curricular activities and research projects related to surgery | 3.29 (1.15) | 3.14 (1.01) | p=0.614 |
